# Analysis of the mitochondrial subproteome of the human cell line AGE1.HN – a contribution to a systems biology approach

**DOI:** 10.1186/1753-6561-5-S8-P86

**Published:** 2011-11-22

**Authors:** Eva Schräder, Sebastian Scholz, Volker Sandig, Raimund Hoffrogge, Thomas Noll

**Affiliations:** 1 Institute for Cell Culture Technology, University of Bielefeld, Bielefeld, Germany; 2 ProBioGen AG, Berlin, Germany

## Background

In Systems Biology approaches a good amount of reliable data is essential for effective modelling of distinct biological processes. Hence the focus of the SysLogics-Project was on modelling of the central metabolism using different functional genomic techniques. For displaying the involved proteins, it is crucial to enrich them using subproteomic fractions. As many enzymes being involved in central metabolism are located in the mitochondria, we investigated the expression dynamics of mitochondrial proteins during batch cultivations of human AGE1.HN cells (ProBioGen, Berlin, Germany).

## Materials and methods

### Cultivation and isolation of mitochondria

Cultivations were performed as batch-processes with chemically-defined and animal-component-free media 42-MAX-UB (Teutocell, Bielefeld, Germany) with addition of 5 mM glutamine. The processes were performed in a 20 L-stainless steel vessel (Sartorius-Stedim, Germany). After disintegration of cells by addition of glass beads (0,1 mm diameter) and vortexing, the isolation of mitochondria was performed with sucrose density-centrifugation in an ultracentrifuge (Beckman Coulter, Krefeld, Germany). Protein extraction from mitochondria was performed with lysis-buffer, which contains Tris-HCl, NaCl, EDTA, SDS, PMSF and NP40.

### Proteomics

The first dimension of 2D-electrophoresis was performed with Ettan IPGPhor3-system*, using pH 3-11 (NL) IPG-strips*. For generating the mitochondrial master-map, six gels with 0.45 mg protein extract were prepared. For DIGE-approaches 0.15 mg were applied, each sample with four replicates. Second dimension was accomplished with Ettan Dalt six Electrophoresis System*. 2DE-gels with DIGE-staining were processed with Ettan Dige Imager*. Software evaluation was implemented with Delta2D (Decodon, Germany).

After isolation and tryptic digest protein-identification was performed with MS/MS using MALDI-ToF/ToF (UltrafleXtreme, Bruker, Germany), followed by database-searching.

*(GE Healthcare, Sweden)

## Results

For proteomic analyses, mitochondria were isolated and successfully confirmed by staining with Rhodamine 123, a mitochondria-specific fluorescent dye. 2DE-gels of mitochondrial fraction led to 519 separated spots. 280 proteins could be reliably identified, out of which more than 50 % are exclusively mitochondrial. This 2D-proteome map is our basis for approaches of analysing different protein expression in mitochondria.

**Figure 1:**
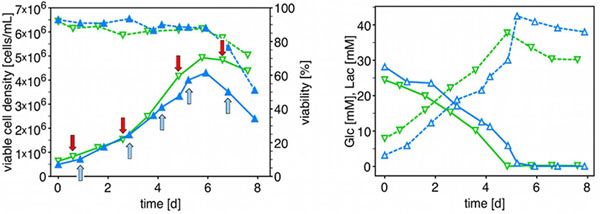
**a** (left): VCD (solid) and viability (dashed) during batch process 1 (open squares) and 2 (closed triangles) **Fig. 1b** (right): Concentrations of glucose (solid) and lactate (dashed) during batch process 1 (open squares) and 2 (closed triangles).

Two standardised batch cultivations in a 20 L-stirred tank stainless steel vessel were carried out with daily sampling for metabolomics, transcriptomics, metabolic flux and proteomics, in order to generate reliable and comparable samples. Batch cultivation in 20 L-scale of AGE1.HN.AAT shows cell densities and duration as expected. Glucose- and lactate-concentrations developed also as estimated in standard batch culture (see fig. 1a+b).

Subproteomic DIGE-analysis of mitochondrial proteins in those 20 L-batch cultivations resulted in 114 proteins that were significantly differently expressed in the first and 206 proteins in the second process during cultivation time (p < 0.05, factorial Anova). More than 40 proteins were found to be significantly regulated in both experiments. DIGE-approaches of mitochondrial fraction of both batch cultivations led to expression profiles of identified proteins. Four examples of expression profiles of typical proteins of TCA and respiratory chain are shown below in table 1.

**Table 1:** Examples of expression profiles of TCA and respiratory chain enzymes. Expression normalised on sample point 1.

	ZE1 (2,6 d)	ZE1 (4,8 d)	ZE1 (6,5 d)	ZE2 (2,9 d)	ZE2 (4,2 d)	ZE2 (5,3 d)	ZE2 (6,9 d)
Pyruvate dehydrogenase E1a	1,53	1,6	0,83	1,51	1,56	1,09	0,87
L-lactate dehydrogenase B chain	0,95	0,87	5,18	0,98	1,53	2,4	3,26
Dihydrolipoamide S-succinyltransferase	2,37	2,18	1,2	2,06	1,92	1,73	0,89
Electron transfer flavoprotein alpha	2,57	2,24	1,38	2,29	1,88	1,99	1,45

Apart from these examples it was possible to observe important biological processes with our 2DE-approach, as for instance:

- tricarboxylic acid cycle

- respiratory chain

- anti-apoptosis and apoptosis

- signal transduction

- chaperone/protein folding and processing

- fatty acid-/lipid-metabolism

- amino acid-degradation

- inhibition of DNA-synthesis

- ketone-metabolism

- protein-biosynthesis

- redox-regulation

- ubiquinone-biosynthesis

Hence, we see this data as a valuable contribution to the Systems Biology approach for modelling central metabolism events.

## Conclusions

Reproducible methods for isolation of mitochondrial proteins from cultured cells for further analysis, such as proteomic 2DE-approaches, have been established. DIGE-analysis of mitochondrial subproteome of AGE1.HN.AAT cells result in the identification of more than 280 identified proteins, of which more than 40 are regulated during batch cultivation. Proteomics data in conjunction with the other "omics"-results offer a promising basis for characterisation of central metabolism during Systems Biology approaches.

